# Gluten Intolerance and Its Association With Skin Disorders: A Narrative Review

**DOI:** 10.7759/cureus.44549

**Published:** 2023-09-01

**Authors:** Vaibhav Vats, Pallavi Makineni, Sarah Hemaida, Anum Haider, Sachin Subramani, Navjot Kaur, Amna Naveed Butt, Renee Scott-Emuakpor, Mohammad Zahir, Midhun Mathew, Javed Iqbal

**Affiliations:** 1 Internal Medicine, Smt. Kashibai Navale Medical College and General Hospital, Mumbai, IND; 2 Medicine, All India Institute of Medical Sciences, Bhubaneswar, Bhubaneswar, IND; 3 Physician, Istanbul Okan University, Istanbul, TUR; 4 Internal Medicine, Bahria University Medical & Dental College, Karachi, PAK; 5 Medicine, ESIC Medical College Gulbarga, Gulbarga, IND; 6 Medicine, Government Medical College, Amritsar, Amritsar, IND; 7 Medicine/Internal Medicine, Allama Iqbal Medical College, Lahore, PAK; 8 Dermatology, University of Miami Miller School of Medicine, Miami, USA; 9 Medicine, Ayub Medical College, Abottabad, Abottabad, PAK; 10 Internal Medicine, Pennsylvania Hospital, Philadelphia, USA; 11 Neurosurgery, Mayo Hospital, Lahore, PAK

**Keywords:** dermatology, celiac disease (cd), skin disorders, gluten-free diet, gluten intolerance

## Abstract

Gluten sensitivity is defined as a chronic intolerance to gluten ingestion in genetically predisposed individuals. The etiology is thought to be immune-mediated and has a variable dermatologic presentation. Celiac disease (CD) is one of the most common forms of gluten intolerance and encompasses a wide range of extra-intestinal pathology, including cutaneous, endocrine, nervous, and hematologic systems. Psoriasis, another long-term inflammatory skin condition, has been linked to significant symptomatic improvement with a gluten-free diet (GFD). Palmoplantar pustulosis (PP), a variant of psoriasis, and aphthous stomatitis, which causes recurrent oral ulcers, have also exhibited beneficial results after the dietary elimination of gluten. In addition to this, dermatitis herpetiformis (DH), another immune-mediated skin disorder, is genetically similar to CD and has, therefore, shown tremendous improvement with a GFD. Another highly prevalent long-term skin condition called atopic dermatitis (AD), however, has revealed inconsistent results with gluten elimination and would require further research in the future to yield concrete results. Hereditary angioedema (HA) has shown an association with gluten intolerance in some patients who had symptomatic benefits with a GFD. Similarly, vitiligo and linear IgA bullous dermatosis have also shown some clinical evidence of reversal with a GFD. On the contrary, rosacea enhances the risk of developing CD. This narrative review emphasizes the potential impact of gluten intolerance on different cutaneous conditions and the potential therapeutic effect of a GFD on various symptomatic manifestations. There is a need for additional clinical and observational trials to further expand on the underlying pathophysiology and provide conclusive and comprehensive recommendations for possible dietary interventions.

## Introduction and background

The term gluten intolerance describes a spectrum of enduring immune-mediated hypersensitivity to ingested gluten within genetically susceptible populations [1]. More than 90% of cases exhibit positive human leukocyte antigen (HLA)-DQ2 (DQA1/DQB1*2) testing [2,3]. The primary explanation centers on suboptimal immune tolerance within genetically predisposed groups, coupled with heightened permeability of the intestinal wall [4,5]. This combination potentially sets the stage for a T-cell-mediated immune reaction within the lamina propria and gut epithelium [3].

Although widely prevalent, celiac disease (CD) often presents minimal to virtually inconspicuous gastrointestinal symptoms. It frequently comes under suspicion due to its comparable immune-mediated presentation and extra-intestinal manifestations, including those involving the thyroid, skin, nervous system, and blood [6]. Chronic lymphocytic (Hashimoto thyroiditis) represents the most frequent association, followed by various skin conditions [6].

In essence, approximately 60% of patients diagnosed with CD and concurrent thyroid disorders exhibit at least one of these dermatological manifestations, encompassing vitiligo, psoriasis, alopecia, stomatitis, atopic dermatitis, and systemic lupus erythematosus (SLE), among others [4]. Humbert et al. have proposed a classification of distinct cutaneous pathologies linked to CD, categorizing them into allergic, autoimmune, inflammatory, and miscellaneous groups [7]. The prevailing complaint is pruritus. Despite the diverse array of cutaneous findings, most patients report a noteworthy improvement after eliminating gluten from their diets [4]. Poon et al. also noted the consistent presence of C3 deposits at the dermal-epidermal junction in a majority (82%) of the cases studied [8].

Despite the accumulating evidence connecting these skin findings to CD, our understanding of their relationship with gluten and precise underlying mechanisms remains limited. This review focuses on the most commonly associated dermatological manifestations of CD, their prognosis, and the documented response to gluten-free diets.

## Review

Immunopathogenesis of skin conditions with gluten intolerance

The diagnosis of non-celiac gluten sensitivity (NCGS) is increasingly associated with a notable predilection toward skin-related disorders. Consequently, a focused investigation into the immunopathological aspects of NCGS, particularly those related to skin lesions, has been undertaken. In a study involving 17 subjects diagnosed with NCGS and exhibiting nonspecific cutaneous manifestations, skin samples were obtained for immunological assessment. Results indicated that the median age of the subjects was 36, with 76% being females. These subjects displayed intensely pruritic cutaneous changes on the extensor areas of the extremities, resembling eczema, psoriasis, or dermatitis herpetiformis. Pathological examination of skin samples revealed deposits of C3, along the dermo-epidermal junction in a microgranular pattern. C3, a pivotal protein in the immune system, functions as a front-line defense against certain diseases involving the body's innate immune response [9].

In another study, excessive production of Th2-related cytokines, such as interleukin (IL)-4 and IL-5, was identified in the serum of patients with dermatitis herpetiformis, both in their skin and blood circulation [10]. Diagnosis of dermatitis herpetiformis hinges on the accumulation of immunoglobulin IgA in the papillary dermis. Distinctive antigenic markers, namely, epidermal TG3 and tissue TG2, differentiate affected sites, targeting IgA deposits in the skin and small bowel mucosa. Although DH may be asymptomatic, active coeliac disease in the gastrointestinal tract leads to the formation of IgA TG3 antibody complexes that localize to the skin [11].

Psoriasis

Psoriasis, a chronic skin condition, exerts systemic impacts on cardiac, metabolic, and musculoskeletal systems. Its pathophysiology primarily revolves around immunological processes, notably involving IL-17, IL-23, and TNF-α [12]. Genetic and autoimmune factors are also intertwined with the disease process [13].

Symptoms: Typical symptoms encompass pruritus, burning, bleeding, and exudation, often accompanied by psychological conditions such as depression [14]. Immune targets have been a cornerstone of therapy, although dietary modifications have shown intriguing outcomes [12,15].

Clinical studies: A Russian study revealed that 14% of psoriasis patients and 2% of controls displayed elevated anti-gliadin IgA antibodies (AGA). Of these, 5% exhibited strong positive antibodies. After implementing a gluten-free diet (GFD) and monitoring for 12 months, the study demonstrated improvement in psoriatic skin lesions and severity scores in AGA-positive patients. This suggests the potential benefits of a GFD for psoriasis patients with positive AGA antibodies [16]. Another study showed that induction of GFD for three months led to a significant reduction in psoriasis severity scores among AGA-positive patients, even those with normal duodenal biopsies. However, AGA-negative patients showed no improvement, and a more pronounced decrease in serum eosinophilic cationic protein was observed in AGA-positive patients [17].

Treatment: Dietary modifications, including GFD and weight reduction, are recommended in conjunction with medical management, alongside potential supplements such as vitamin D for psoriasis [15].

Palmoplantar Pustulosis

Pathophysiology: Recently, dermatological conditions, such as palmoplantar pustulosis and gluten intolerance, have gained crucial attention because of how greatly these conditions may impact the diagnosed patients' quality of life. While these two conditions may appear independent of each other, emerging evidence suggests a potential link between them [18]. Palmoplantar pustulosis is a chronic skin condition that predominantly affects the palms of the hands as well as the soles of the feet. 

Symptoms: The emergence of minuscule, pus-drenched blisters in the abovementioned areas are the main physical characteristics that subjectively define this ailment [18]. An individual experiencing a palmoplantar pustulosis flare-up may exhibit symptoms, such as thickened skin, skin redness, or skin peeling, accompanied by unbearable itchy, and burning sensations along the inflamed regions [18]. Furthermore, palmoplantar pustulosis is a variant of psoriasis, yet its pathophysiology remains inconclusive within the medical community, and little is known about the genesis of the condition. 

Clinical studies: A study involving 123 individuals with palmoplantar pustulosis examined IgA antibodies against gliadin and tissue transglutaminase following a GFD. The results indicated that those with IgA antibodies could experience symptom alleviation through a GFD [18]. Another study involving psoriasis patients without CD demonstrated clinical improvement after adhering to a strict GFD for at least 90 days [19]. Although the specific mechanisms connecting palmoplantar pustulosis and gluten intolerance require further investigation, shared immune system dysregulation components between palmoplantar pustulosis and psoriasis are speculated to play a role [19,20].

In conclusion, recognizing the potential link between dermatological disorders and seemingly unrelated conditions such as gluten intolerance is essential for comprehensive patient care and could aid in understanding the underlying causes of these conditions [21].

Aphthous Stomatitis

Aphthous stomatitis, commonly known as canker sores, is characterized by the presence of painful mucosal ulcers in nonkeratinized areas of the mouth and throat [22].

Pathophysiology: Although no single identifiable cause has been pinpointed, the etiology of aphthous stomatitis is believed to be multifactorial [23]. This condition has been associated with autoimmune disorders, such as Behcet's disease, inflammatory bowel disease, CD, and SLE, as well as dermatological disorders, malnutrition (e.g., deficiencies in vitamin B12, folate, and iron), infections (e.g., HIV), and certain drugs (e.g., methotrexate). Local trauma, such as accidentally biting the tongue, is also a common trigger [23,24]. T-cell-mediated immune dysfunction and the secretion of cytokines such as TNF-alpha play a significant role in the development of aphthous ulcers [25].

Symptoms: Aphthous ulcers are characterized by their round to oval shape, resembling craters with yellowish-gray bases and erythematous margins on efflorescence [23]. These ulcers can manifest in different forms, including minor ulcers (the most common), major ulcers, and herpetiform ulcers [26].

Clinical studies: Research by Shakeri et al. in Iran examined 247 patients with recurrent aphthous ulcers. A subset of patients (2.83%) showed a significant improvement in symptoms over six months after adopting a strict GFD. This improvement was observed even in cases that did not respond to conventional anti-aphthae treatment [27]. Another study by Ferguson et al. in Birmingham reported similar results, indicating gluten sensitivity as an underlying cause of aphthous ulcers [28]. A study by Wray on 20 patients with recurrent aphthous stomatitis, all on a GFD, found favorable responses in 25% of patients, suggesting gluten sensitivity even without enteropathy [29]. A meta-analysis showed that patients with CD had a significantly higher frequency of aphthous stomatitis compared to healthy subjects (OR=3.79, 95% CI=2.67-5.39) [30]. Additionally, Ferguson et al. recommended screening for deficiencies in folate, B12, and iron in patients with recurrent aphthae, with jejunal biopsy reserved for cases showing evidence of malabsorption [31].

Treatment: Management of aphthous stomatitis focuses on symptom relief and includes the use of topical corticosteroids, antimicrobials, and anesthetics [32]. The studies mentioned above suggest that screening for vitamin deficiencies and serological markers could enhance the evaluation and management of aphthous ulcer treatment [29-31].

Cutaneous Small Vessel Vasculitis (CSSV)

The literature has infrequently documented the connection between CD and cutaneous vasculitis [33-37]. Leukocytoclastic vasculitis is a histopathologic diagnosis given to CSSV involving arterioles, capillaries, and venules. This condition is characterized by the accumulation of an inflammatory infiltrate, primarily composed of neutrophils, with associated fibrinoid necrosis and disintegrated nuclear fragments around the cutaneous small vessels [38].

Pathophysiology: The etiology of CSSV mainly includes viral or bacterial infections (20%), inflammatory diseases (15%-20%), various drug reactions (10%-15%), malignancies (5%), and idiopathic origins for the rest [39,40]. The pathogenesis involves the deposition of immune complexes around the small vessel walls, triggering and activating the complement system. Neutrophils are recruited, leading to vessel wall injury and extravasation of red blood cells, fibrin, and serum [41].

Symptoms: Cutaneous manifestations typically appear on the lower extremities and encompass palpable purpura, nodules, skin vesicles, bullous skin lesions, and livedo reticularis [42].

Clinical studies: In a case report by Meyers et al., a 38-year-old female with a 20-year history of CD developed purpuric painful nodules in the lower extremities. Skin biopsy confirmed leukocytoclastic vasculitis. The patient adhered to a strict GFD, leading to complete resolution of skin lesions within four months [36].

In CD patients, increased intestinal permeability to exogenous antigens arises from bowel wall damage [43]. Additionally, endogenous antigens released from the injured small bowel mucosa can lead to autoimmune sensitization [44,45]. Circulating immune complexes from these antigens may deposit in the skin due to compromised phagocytic function of the reticuloendothelial system, leading to small vessel wall damage via complement system activation and neutrophil recruitment, ultimately causing cutaneous vasculitis. A skin biopsy taken from the earliest, most symptomatic skin lesion serves as the gold standard for diagnosing cutaneous vasculitis. Direct immunofluorescence (DIF) to detect immunoglobulins holds diagnostic and prognostic value [38,46]. Basic blood work, including complete blood count, complete metabolic panel with liver function tests, erythrocyte sedimentation rate, and urinalysis, should be conducted if systemic involvement is not suspected. Further extensive testing is required if systemic involvement is suspected [38,42]. Approximately 90% of CSSV cases are self-limited, resolving spontaneously within weeks to months.

Treatment: The adoption of a strict GFD has proven highly beneficial, as reported by Marsh et al. and Pulido-Pérez et al. [44,45] and is recommended in various literature reviews [36,44]. The treatment of leukocytoclastic vasculitis often involves corticosteroids. Notably, cutaneous vasculitis incidence appears higher in patients with poorly controlled CD [33,36,47].

*Alopecia Areata* *(AA)*

AA is an autoimmune condition causing non-scarring hair loss. Prevalent in approximately 2% of the population, AA's occurrence spans various age groups and both sexes [48].

Clinical presentation: AA's clinical presentation varies, from well-defined areas of hair loss to complete body hair loss, including scalp hair [49]. Nail involvement, characterized by pitting and friability, affects around 10-20% of cases [49]. Disease severity and age at onset dictate prognosis [50].

Pathophysiology: While the exact mechanism of AA remains unclear, diminishing immune tolerance of hair follicles is believed to contribute to their destruction and promote inflammatory pathways [50]. These changes may manifest as dystrophic hair follicles with yellow dots on trichoscopy.

Clinical studies: AA, like other autoimmune diseases, shows associations with SLE, rheumatoid arthritis, autoimmune thyroiditis, Addison's disease, and vitiligo [51]. An association between AA and CD was first outlined by Corazza et al. in 1995 [52]. This link has been supported by subsequent studies, with prevalence estimates ranging from 1:85 to 1:116 [53,54]. Interestingly, a higher occurrence of AA and vitiligo is reported in dermatitis herpetiformis patients compared to the general population [55]. Elevated prevalence of anti-gliadin antibodies is also linked to extensive diseases such as alopecia universalis [56]. Ertekin et al. found that 41.7% of pediatric AA patients tested positive for IgA anti-tG Ab, and these patients exhibited total villous atrophy (Marsh type-IIIc) on intestinal biopsy. Eliminating gluten restored hair growth, suggesting CD screening for pediatric AA patients. However, a 2016 study by Mokhtari et al. found no significant difference in CD serological markers between case and control groups (p=0.35), suggesting the need for alternate CD detection methods [56].

Treatment: Despite limited evidence, a GFD trial is recommended for refractory AA cases [57]. Clinical improvement in CD-related autoimmune conditions upon adopting a GFD is attributed to immune homeostasis restoration [54]. Many patients on a GFD experience normal hair regrowth without further recurrences, despite the usual recurrence pattern observed in alopecia areata [58].

Dermatomyositis (DM)

DM is an autoimmune connective tissue disorder primarily affecting the skin and skeletal muscles [59]. It is also associated with other diseases, such as inflammatory myopathies (IM), categorized into DM, polymyositis (PM), and sporadic inclusion body myositis (sIBM) based on histopathology [60]. While this association is documented in children, its significance in adults remains to be clarified [61].

Symptoms: Symptoms include various skin findings, such as heliotrope rash, Gottron’s papule, and Holster sign. Limb weakness, especially proximal, is prominent. Investigations reveal elevated CPK levels, EMG changes, and muscle biopsy indicating peri fascicular atrophy [59,62].

Clinical studies: In a study examining the link between CD and IM, IgA class AGA antibodies were found in 11% of DM patients, 44% of PM patients, and 100% of sIBM patients. Notably, these antibodies were more frequently associated with sIBM than DM [60]. A case reported a 40-year-old female with CD-associated DM. Symptoms of both conditions resolved with a GFD, emphasizing the importance of testing for CD in DM patients, even without GI symptoms [61].

SLE

Pathophysiology: SLE (lupus) is a chronic autoimmune disease characterized by hyperactive immune responses targeting healthy cells and tissues [63]. While lupus affects various organs, it particularly impacts the integumentary system, leading to significant immune-mediated damage [64]

Symptoms: Prominent symptoms include a butterfly-shaped rash on the face, loss of appetite, fever, mouth ulcers, and painful, swollen joints [65]. Research has shown an association between lupus and CD, with some studies reporting a higher prevalence of lupus in individuals with biopsy-verified CD [66, 67]. However, further research is needed to fully understand the relationship [66].

Clinical studies: A recent study examining the correlation between SLE and adult CD involved the evaluation of 246 patients diagnosed with both conditions. Among these patients, six individuals (2.4%) with CD and lupus experienced a diagnosis of lupus at least a decade or 10 years after being diagnosed with CD. This delayed lupus diagnosis occurred despite the normalization of small intestinal biopsies following adherence to a GFD. These findings suggest a potentially higher prevalence of SLE, which could be identified through an extended clinical follow-up period among biopsy-defined CD patients [68].

In a subsequent study conducted by Rensch et al. involving 103 SLE patients to assess the prevalence of CD, it was found that 24 patients (23.3%) tested positive for IgA and IgM antigliadin antibodies. However, none of these 24 patients exhibited endoscopic or histologic findings consistent with CD, indicating a notable false-positive rate for antigliadin antibodies. The study concluded that there is no significant association between CD and lupus [67].

Lastly, in the research study titled "Increased Risk of Systemic Lupus Erythematosus in 29,000 Patients with Biopsy-Verified Celiac Disease," the case presented demonstrates that individuals diagnosed with CD faced a noteworthy threefold increase in the risk of developing SLE compared to the general population. However, the study consistently highlights that the absolute risks of developing lupus within this cohort remain low [68].

Treatment: Based on the existing literature, one is able to conclude that the best treatment option for individuals living with dermatologic manifestations brought about by a diagnosis of SLE may be similar to that of an individual discord with CD or gluten sensitivity, a GFD [68].

Undifferentiated Connective Tissue Disorder (UCTD)

UCTD refers to systemic autoimmune conditions with one or more clinical manifestations of definite connective tissue disease (CTD) lasting at least three years, accompanied by positive ANA results. UCTD shares clinical and serological features with established CTDs while not meeting any current classification criteria [69]. 

Pathophysiology: Immunoregulatory mechanisms are believed to play a role in the pathogenesis of UCTD. A study by Szodoray et al. revealed an association of UCTD with decreased natural regulatory T-cells. A further decrease was associated with disease activity, progression into a differentiated CTD, and poor prognosis [70].

Symptoms: The presentation of patients with UCTD can vary with a wide range of symptoms, with arthralgia and cutaneous features being the most common [71].

Clinical studies: Conti et al. prospectively and retrospectively studied 52 patients diagnosed with undifferentiated CTD after subdividing them into different CTD-like subgroups. An endoscopic biopsy and histological examination were done in six patients positive for CD serology (anti-gliadin, anti-endomysium, and anti-TTG antibodies). Four out of the six patients showed villous atrophy, with 83% of celiac patients showing a scleroderma-like phenotype. Symptoms of fever and myalgia regressed early following a GFD suggesting early recommendation of GFD to all patients with UCTD and GSE [72]. 

Rosato et al. investigated positive celiac serology in 50 patients with systemic sclerosis (SSc). Duodenal histology was studied in four out of five patients positive for serology to diagnose CD according to Marsh/Oberhuber classification. All four patients studied had positive histological findings. The study showcased a significant occurrence of CD in individuals with SSc, implying the need for regular CD screening in SSc patients [73]. Nisihara et al. conducted a case-control study involving 105 individuals with scleroderma (34.2% diffuse scleroderma and 56.2% limited scleroderma) and 97 volunteers in Brazil. The study revealed no elevated occurrence of anti-endomysial antibodies among individuals with scleroderma, concluding the unnecessity of routine screening for CD without a clear indication. The variation might be explained by variation in genetic makeup and the proportion of generalized SSc form under investigation, necessitating broader multicenter studies for additional insights [74].

Urticaria

Urticaria (hives) is a skin condition characterized by itchy, raised, and red welts that can appear and disappear over hours or days [75].

Pathophysiology: The underlying pathophysiology involves various triggers, including allergic reactions, infections, medications, and autoimmune conditions, such as CD [75]. CD, an autoimmune condition caused by gluten ingestion, leads to immune system dysregulation and inflammation in the small intestine, causing damage to the villi responsible for nutrient absorption [76]. The connection between urticaria and CD lies in the shared autoimmune nature and potential immune system dysregulation. CD can contribute to urticaria through increased immune activation, mast cell activation, potential cross-reactivity to non-gluten proteins, and alterations in the gut microbiota influencing skin conditions [76].

Symptoms: Clinical symptoms of urticaria include raised welts of varying size and shape, intense itching, redness, swelling, transient nature, burning or stinging sensations, and sometimes angioedema. In severe cases, systemic symptoms like difficulty breathing, dizziness, nausea, vomiting, or anaphylaxis may occur [77].

Clinical studies: Here, we highlight key findings from relevant studies, shedding light on the association between gluten intolerance and urticaria. One compelling case study by Hautekeete et al. illustrated a significant improvement in chronic urticaria symptoms after implementing a GFD. The patient experienced a notable weight gain of 7 kg over several months and observed a gradual amelioration of urticaria after two months, ultimately leading to its complete disappearance after three months on the GFD. Subsequent small bowel biopsies demonstrated partial regeneration of villi, reinforcing the connection between gluten intolerance and chronic urticaria [78]. The prevalence of urticarial rash has been investigated in various autoimmune diseases, including CD.

A study conducted by Kolkhir et al. revealed that urticarial rash was notably higher in eosinophilic granulomatosis with polyangiitis, rheumatoid arthritis (RA), autoimmune thyroid disease, SLE, and CD patients. CD patients exhibited a rate of urticarial rash above 1.5%, emphasizing the potential link between these conditions [79]. Moreover, patients with chronic spontaneous urticaria, particularly adult females with positive family history and genetic predisposition for autoimmune disorders, were found to be more vulnerable to developing autoimmune diseases. As such, a diligent screening for signs and symptoms of autoimmune diseases is crucial in this subgroup of patients [79]. The resolution of symptoms in a CD patient on a GFD further supports the correlation between gluten intolerance and urticaria.

Pedrosa et al. reported that, after seven months on a GFD, the patient's anemia improved, transaminase levels decreased, iron metabolism normalized, and cold urticaria episodes did not recur. These observations were consistent with the normalization of IgA anti-tissue transglutaminase (TTG) titers, confirming the benefit of a GFD in the reduction of the disease [80].

Atopic Dermatitis

Atopic dermatitis is a prevalent, chronic skin condition characterized by a compromised skin barrier, leading to persistent inflammation, affecting up to 20% of children and 10% of adults worldwide [81].

Pathophysiology and clinical symptoms: The fundamental pathophysiology of AD involves a compromised skin barrier, resulting in moisture loss and creating an entry point for bacteria and other pathogenic microorganisms. This dry skin condition leads to itching, lichenification, susceptibility to infections, and disrupted sleep patterns [82].

Clinical studies: AD has a multifaceted etiology involving environmental, genetic, and immunologic factors [83]. Notably, the potential link between gut health and AD has been extensively discussed. A cross-sectional study involving 169 individuals demonstrated that 51% of participants with atopic dermatitis experienced improvement upon eliminating gluten from their diet [84]. Sur et al. presented a case report about a 32-year-old woman and her two daughters, diagnosed with AD in early childhood, who observed substantial symptom alleviation after adopting a GFD [85]. Additionally, a specific form of gluten sensitivity known as CD is associated with AD. A study focusing on the pediatric population revealed that 45% of children with CD had concurrent AD [82]. Another study indicated a 3.8% prevalence of AD among adult patients with CD [86].

The primary mechanism underlying these findings suggests that gluten consumption triggers inflammation and malabsorption in genetically predisposed individuals, leading to extra-intestinal manifestations, particularly cutaneous inflammatory conditions like AD [87]. Furthermore, the prolamins, which are glutamine/proline-rich proteins present in gluten, are challenging to digest due to their distinctive peptide bonds.

Treatment: Although research has concentrated on peptidases such as POP and PEP, achieving complete gluten hydrolysis often necessitates the combination of different peptidases. Further investigation is required to ascertain their efficacy and safety for therapeutic applications. Enzyme therapy may complement a GFD by mitigating gluten-induced effects resulting from inadvertent consumption, but it should not be regarded as a standalone treatment for CD [88].

Hereditary Angioedema

Hereditary angioedema is a rare genetic disorder characterized by localized tissue edema, swelling, and redness, resulting from increased blood vessel permeability due to vasodilators and pro-inflammatory agents, such as bradykinin and histamine - a condition known as angioedema. Angioedema can either be hereditary or acquired [89].

Pathophysiology: The underlying pathophysiology of this disorder involves a deficiency or functional inactivity of the enzyme C1 inhibitor. This deficiency disrupts several important biochemical mediator pathways, including complement, fibrinolytic, coagulation, and kinin cascades. Consequently, there is an elevated production of bradykinin, contributing to the disease's clinical manifestations [90].

Symptoms: Symptomatically, patients with hereditary angioedema present with episodic and non-pruritic swelling of the skin and associated tissues in various areas, such as extremities, abdomen, gastrointestinal tract, face, and neck structures [91].

Clinical studies: Clinical studies have suggested a correlation between hereditary angioedema and gluten intolerance [90,92]. A comprehensive study involving 128 patients with hereditary angioedema revealed that 18.1% of them also suffered from gluten intolerance and CD - significantly higher than the normal prevalence of 1.2% for CD. Diagnosis of CD was established using IgA-endomysial antibodies, IgG-endomysial antibodies, IgA-tissue transglutaminase, and IgG-tissue transglutaminase. The study reported that incorporating a GFD into patients' meals and substituting tranexamic acid-based wheat with danazol-based wheat starch alleviated abdominal symptoms. However, the study did not elucidate any potential impact of this dietary intervention on the cutaneous manifestations of hereditary angioedema. It is noteworthy that both hereditary angioedema and CD can present with abdominal symptoms due to their involvement in the gastrointestinal tract. Consequently, the study recommends screening patients with hereditary angioedema for CD if abdominal symptoms persist despite management. Furthermore, patients with CD should undergo screening for hereditary angioedema if they report persistent abdominal symptoms even after initiating a GFD in their treatment regimen [93].

Vitiligo

Vitiligo is a primarily acquired disorder characterized by progressive, autoimmune-mediated loss of melanocytes from the skin. As a consequence, depigmented patches develop on the affected areas, appearing lighter in shade than the surrounding skin [94]. According to a report, vitiligo affects 0.5-2% of the global population [95].

Pathophysiology: To date, the specific causes of vitiligo remain elusive, and no definitive environmental associations have been identified. Several theories have been proposed to elucidate the etiology behind this condition. The most widely accepted theory is the autoimmune theory mentioned earlier [94]. Furthermore, studies have indicated that individuals with vitiligo are at a relatively higher risk of developing other autoimmune disorders, such as pernicious anemia, diabetes mellitus, and thyroid diseases, compared to the general population [96-100].

Clinical studies: Two case reports have suggested a potential relationship between vitiligo and a GFD. The first case report [101] describes a 9-year-old girl with both CD and vitiligo, who exhibited significant repigmentation three years after adopting a GFD. The repigmentation persisted for seven years after initiating the diet. The second case report [102] outlines the improvement and repigmentation of a 22-year-old female without CD or gluten intolerance following the adoption of a GFD. These cases hint at a connection between a GFD and vitiligo, even in patients without CD. Hence, exploring the effects of a GFD on vitiligo patients is deemed worthwhile.

Moreover, two studies have explored the relationship between vitiligo and IgA antibodies associated with CD. In the initial cross-sectional study, serology testing of 198 vitiligo patients did not detect any antibodies [103]. However, a more recent case-control study demonstrated a potential link between vitiligo and CD. Of the 64 vitiligo patients, two were found positive for EMA-IgA and tTG IgA antibodies related to CD, while all 64 control participants tested negative for these antibodies [104]. These findings suggest a possible connection between vitiligo and a gluten diet.

Rosacea

Rosacea is defined as a skin condition primarily prevalent among light-skinned individuals, affecting the mid-facial area, including the nose, forehead, and cheeks. It is characterized by erythrogenic (redness-inducing) and inflammatory features, often exhibiting a chronic nature. While rosacea is more commonly observed in light-skinned individuals, several studies have documented its occurrence in people of Latin, African, or Asian descent [105,106]. While rare, rosacea can be more severe in males and more prevalent in females. The onset of the disease typically occurs after the age of 30 [107-109].

Pathophysiology: The underlying mechanisms behind rosacea remain unclear and not fully understood. However, the condition has been associated with various triggering and exacerbating factors, including transient temperature changes, physical exhaustion, strenuous activity, exposure to UV rays, alcohol consumption, and certain microorganisms, such as Bartonella quintana, Helicobacter pylori, and Demodex species [110,111]. Rosacea manifests its symptoms through immune system modulation and heightened vascular sensitivity. Specifically, toll-like receptors 2 (TLR-2), TRP ion channels, and specific cytokines that play crucial roles in inflammatory responses are implicated [112]. It is important to note that individuals with rosacea often experience emotional and social distress due to the visible facial manifestations associated with the condition, leading to anxiety and depression [113].

Clinical studies: A case-control study [114] involving 6759 rosacea patients revealed significantly elevated rates of diabetes mellitus type 1 and CD, particularly among females. Furthermore, a higher prevalence of CD was noted in rosacea patients compared to control subjects [115]. These findings establish an association between gluten intolerance (CD) and rosacea, suggesting that implementing a GFD might offer relief for rosacea patients. This potential connection warrants exploration through trials involving rosacea patients.

Linear IgA Bullous Dermatosis (LABD)

Linear IgA is a rare autoimmune blistering disease, which can sometimes be triggered by medications. It is characterized by the deposition of linear IgA at the dermo-epidermal junction, occurring in both the epidermis and mucous membranes such as the oral and vaginal mucosa, as well as the conjunctivae. This condition can affect individuals of all ages, including both adults and children. In children, it is referred to as chronic bullous dermatosis of childhood, and its causes and symptoms are similar to those of bullous pemphigoid and dermatitis herpetiformis. Notably, over 50% of drug-induced cases in adults are attributed to vancomycin [116].

Clinical presentation: While children commonly experience lesions on the face and perineum, the disease can manifest on any part of the body, including the extremities, abdomen, feet, and scalp. In adults, the trunk is often affected, with the head, face, and limbs also susceptible. These lesions typically present as a skin eruption that can cause discomfort. Mucosal involvement is observed in both age groups, though milia are rare [117]. Several HLAs, including HLA-B8, HLA-DR3, HLA-DQ2, and HLA-cw7, have been associated with the incidence of this condition [118].

Pathophysiology: Distinguishing linear LABD from dermatitis herpetiformis involves the presence of IgA anti-basement membrane antibodies in the bloodstream. These antibodies bind to the 97-kD protein of BPAG2 (bullous pemphigoid antigen 2), which is expressed in the lamina lucida of human skin [119].

Clinical studies: GFDs have shown efficacy in managing skin blisters and enteritis associated with dermatitis herpetiformis. However, minor mucosal intestinal abnormalities are less commonly linked to linear IgA disorders. Interestingly, blisters do not consistently improve with gluten restriction. A study involving a patient diagnosed with linear LABD and enteritis investigated the potential relationship between the two conditions. Through continuous monitoring and analysis of jejunal and skin biopsy samples, it was observed that a GFD led to the resolution of both jejunal anomalies and skin lesions. Reintroduction of gluten resulted in the reappearance of these issues. This study suggests that gluten restriction could alleviate the symptoms of linear LABD when underlying gluten-sensitive enteritis is present [120].

Treatment: Since its recognition in the 1970s, significant progress has been made in treating linear LABD. Oral dapsone remains the preferred medication, demonstrating remarkable effectiveness by clearing visible lesions within two to three days. Dapsone is often administered at low doses to effectively prevent infection. Close monitoring is crucial to detect signs of hypersensitivity reactions, neutropenia, liver disease, peripheral nerve damage, and kidney issues in individuals using dapsone. In some cases, initial control of the condition may require additional administration of oral corticosteroids [121]. Antibiotics such as tetracycline, dicloxacillin, and trimethoprim-sulfamethoxazole have shown potential in managing symptoms, even though the exact infectious source of the disease remains unidentified. In cases of vancomycin-induced LABD, discontinuing vancomycin and substituting it with an alternative antibiotic, such as doxycycline, can enhance treatment, providing comparable microbiological coverage. Additionally, adjunctive treatment involving daily intake of 15 mg of nicotinamide has demonstrated promise [122].

Dermatitis Herpetiformis

Dermatitis herpetiformis is an immune-mediated skin disorder that manifests as a pruritic papulovesicular rash on extensor surfaces of the body and has a strong association with gluten sensitivity. It is also linked to CD. DH has the presence of deposits of IgA in the papillary dermis, which are granular in appearance [123]. 

Pathophysiology: Both of these conditions occur in individuals who are gluten-sensitive and share the same human leukocyte antigen types, DQ8 and DQ2. Many of these patients exhibit small bowel alterations, including villous atrophy and increased intraepithelial lymphocytes. Autoantibodies against tissue transglutaminase (tTG) are also commonly found [124]. Diagnosis is often aided by DIF. Serologically, anti-epidermal transglutaminase (TG3) antibodies are crucial for diagnosis, while anti-tissue transglutaminase (TG2) antibodies can serve for both diagnosis and disease monitoring [125]. DH and CD are both gluten-dependent enteropathies, sharing antibodies against tissue transglutaminase. While the autoantigen in the stomach is tissue transglutaminase, the IgA immune response in DH targets epidermal transglutaminase. A pivotal diagnostic feature of DH is the presence of granular IgA deposition in the papillary dermis [126].

Symptoms: In patients with gluten-sensitive enteropathy, DH manifests as erythematous, excoriated papules that are intensely pruritic, primarily affecting the elbows, buttocks, and knees, and vesicles are infrequent [127]. 

Clinical studies: A prospective study was conducted on 514 patients with dermatitis herpetiformis in 1970. The analysis included all DH patients diagnosed with CD for a minimum of two years. Additionally, 20 patients (4%) had CD before developing DH. The average time for diagnosing both dermatitis herpetiformis and CD was 9.5 years. Prior to DH diagnosis, four patients were on a gluten-containing diet, 10 experienced lapses in GFD adherence, and six strictly adhered to a GFD. Among 19 patients, seven displayed celiac autoantibodies, with five out of the seven exhibiting partial villous atrophy on small intestinal biopsy. The rash was effectively managed with a strict GFD post-DH diagnosis [128]. In a seminar by Rodrigues et al. on CD, it was reported that 90% of patients with dermatitis herpetiformis had an association with gluten-sensitive enteropathy, as observed through endoscopy [129].

Treatment: DH is typically treated with a combination therapy of a GFD and dapsone. Dapsone primarily acts as an anti-inflammatory agent, downregulating neutrophil chemotaxis and reducing the release of prostaglandins and leukotrienes, thus preventing tissue damage. While a GFD can aid in managing both cutaneous and intestinal symptoms, medications such as dapsone and sulfones are effective in addressing skin manifestations only [130].

## Conclusions

Gluten intolerance is characterized by a large variety of clinical presentations with both intestinal and extraintestinal presentations. The relationship between gluten intolerance and dermatitis herpetiformis has been studied in detail, but there is a lack of data on other dermatological conditions such as psoriasis, alopecia, urticaria, etc. In our review, we mainly focused on the various dermatological conditions and tried to identify the relationship and potential mechanism between these skin conditions and gluten intolerance. Screening for CD and gluten sensitivity should be encouraged in patients presenting with dermatological conditions of unknown etiology. An appropriate evaluation of various dermatological conditions could unfold the underlying cutaneous-gastrointestinal connection and ensure that important gastrointestinal disease in patients with skin manifestations is not ignored. Strict adherence to a GFD is the treatment for these dermatological conditions, and this has been well-documented in the literature. With our review, we hope to shed more light on the cutaneous-gastrointestinal connection and to encourage further investigations and develop guidelines for the management of skin manifestations in gluten intolerance.
